# Altered Urinary Amino Acids in Children With Autism Spectrum Disorders

**DOI:** 10.3389/fncel.2019.00007

**Published:** 2019-01-25

**Authors:** Aiping Liu, Wei Zhou, Liuhong Qu, Fusheng He, Hui Wang, Yan Wang, Chunquan Cai, Xiaoge Li, Wenhao Zhou, Mingbang Wang

**Affiliations:** ^1^Shiyan Prevention and Health Care Center of Shenzhen, Shenzhen, China; ^2^Division of Neonatology, Guangzhou Women and Children’s Medical Center, Guangzhou Medical University, Guangzhou, China; ^3^Division of Neonatology, The Maternal and Child Health Care Hospital of Huadu District, Huadu Affiliated Hospital of Guangdong Medical University, Guangzhou, China; ^4^Imunobio, Shenzhen, China; ^5^Xiamen Branch of Children’s Hospital of Fudan University (Xiamen Children’s Hospital), Xiamen, China; ^6^Division of Neurosurgery, Tianjin Children’s Hospital, Tianjin, China; ^7^Tianjin Jinnan Xiaozhan Hospital, Tianjin, China; ^8^Shanghai Key Laboratory of Birth Defects, Division of Neonatology, Children’s Hospital of Fudan University, National Center for Children’s Health, Shanghai, China; ^9^Shanghai Key Laboratory of Birth Defects, Division of Neonatology, Xiamen Branch of Children’s Hospital of Fudan University (Xiamen Children’s Hospital), Children’s Hospital of Fudan University, National Center for Children’s Health, Shanghai, China

**Keywords:** ASD, urinary amino acids, metabolome, LC-MS/MS, children

## Abstract

Autism spectrum disorders (ASD) affect 1% of children. Although there is no cure, early diagnosis and behavioral intervention can relieve the symptoms. The clinical heterogeneity of ASD has created a need for improved sensitive and specific laboratory diagnostic methods. Liquid chromatography-tandem mass spectrometry (LC-MS/MS)-based analysis of the metabolome has shown great potential to uncover biomarkers for complex diseases such as ASD. Here, we used a two-step discovery–validation approach to identify potential novel metabolic biomarkers for ASD. Urine samples from 57 children with ASD and 81 matched children with typical development (TD) were analyzed by LS-MS/MS to assess differences in urinary amino acids and their metabolites (referred to as UAA indicators). A total of 63 UAA indicators were identified, of which 21 were present at significantly different levels in the urine of ASD children compared with TD children. Of these 21, the concentrations of 19 and 10 were higher and lower, respectively, in the urine of ASD children compared with TD children. Using support vector machine modeling and receiver operating characteristic curve analysis, we identified a panel of 7 UAA indicators that discriminated between the samples from ASD and TD children (lysine, 2-aminoisobutyric acid, 5-hydroxytryptamine, proline, aspartate, arginine/ornithine, and 4-hydroxyproline). Among the significantly changed pathways in ASD children were the ornithine/urea cycle (decreased levels of the excitatory amino acid aspartate [*p* = 2.15 × 10^-10^] and increased arginine/ornithine [*p* = 5.21 × 10^-9^]), tryptophan metabolism (increased levels of inhibitory 5-hydroxytryptamine *p* = 3.62 × 10^-9^), the methionine cycle (increased methionine sulfoxide [*p* = 1.46 × 10^-10^] and decreased homocysteine [*p* = 2.73 × 10^-7^]), and lysine metabolism (reduced lysine [*p* = 7.8 × 10^-9^], α-aminoadipic acid [*p* = 1.16 × 10^-9^], and 5-aminovaleric acid [*p* = 1.05 × 10^-5^]). Collectively, the data presented here identify a possible imbalance between excitatory and inhibitory amino acid metabolism in ASD children. The significantly altered UAA indicators could therefore be potential diagnostic biomarkers for ASD.

## Introduction

Autism spectrum disorders affect more than 1% (from 1 in 88 to 1 in 68) of children worldwide (Developmental Disabilities Monitoring Network Surveillance Year 2010 Principal Investegators and Centers for Disease Control Prevention, 2014). Although there is currently no cure, early diagnosis and behavioral intervention can effectively alleviate the symptoms (Dawson et al., 2010). Therefore, there is an urgent need for the development of laboratory tests for early diagnosis of ASD. At present, laboratory diagnosis mainly relies on chromosome microarray analysis (Miller et al., 2010), which has a detection rate of about 10–20% (Carter and Scherer, 2013). Recently developed diagnostic methods based on whole exome/genome sequencing have increased the detection rate to 20–40% (Jiang et al., 2013; Tammimies et al., 2015), but the cost is high. Moreover, ASD displays strong clinical heterogeneity (Betancur, 2011), and a large number of cases may not be diagnosed by genetic methods.

Liquid chromatography-tandem mass spectrometry-based analysis of urine samples has been used successfully to search for biomarkers for a number of complex diseases (Dunn et al., 2011), including ASD (Yap et al., 2010; Ming et al., 2012; Emond et al., 2013; Mavel et al., 2013; Cozzolino et al., 2014; Nadal-Desbarats et al., 2014; Noto et al., 2014; West et al., 2014; Dieme et al., 2015; Gevi et al., 2016; Wang et al., 2016; Lussu et al., 2017). Here, we used this approach to examine UAAs and related metabolites (referred to as UAA indicators) in samples from 57 ASD children and 81 matched typically developing (TD) children in a two-step discovery–validation approach. Our goal was to identify UAA indicators that could potentially be developed as biomarkers for ASD.

## Materials and Methods

### Participants

A total of 57 children with ASD and 82 TD children were recruited for this study. The inclusion and exclusion criteria are detailed in our previous reports (Zhou et al., 2017, 2018). In brief, the inclusion criteria for ASD children were: boys or girls younger than 14 years, diagnosis of ASD according to the criteria in the Diagnostic and Statistical Manual of Mental Disorders V (American Psychiatric Association, 2013), the exclusion criteria for ASD children: diagnosis of other mental illness (e.g., attention-deficit hyperactivity disorder, obsessive compulsive disorder), other neurodevelopmental disorder, genetic metabolic disease, or severe neurological disease; history of brain injury, taking non-essential medications or dietary supplements 72 h prior to and during the specimen collection. The TD group consisted of age-matched healthy children. Inclusion criteria for TD children were: healthy, with no mental illness, and age matched with the ASD children, the exclusion criteria were the same as for the ASD children. The study was conducted in two phases. The discovery cohort included 28 ASD children (26 males, 92.8%) aged 5.4 ± 2.4 years (mean ± standard deviation [SD]; range 2–12 years) and 41 TD children (19 males, 73.2%) aged 5.1 ± 2.7 years (range 1–13 years). The validation cohort consisted of 29 ASD children (27 males, 93.1%) aged 5.3 ± 2.2 years (range 2–12 years) and 41 TD children (7 males, 29.2%), aged 5.1 ± 2.5 years (range 2–14 years). Both written and informed consent was obtained from the parents of the participants in this study. The study was conducted in accordance with the Declaration of Helsinki and the study protocol was approved by the ethics committee of Xiamen Children’s Hospital.

### Sample Collection and Derivatization of Urinary Amino Acids

The second urine in the morning were collected in disposable cups, a 15 ml urine sampling tube containing 60 mg of oxalic acid was used for dispensing samples, the samples were frozen at -80°C within 2 h of collection before use. Aliquots of 1 ml urine were mixed with 1 ml 38% hydrochloric acid, sealed under nitrogen, and incubated at 110°C for 21 h. Then, 50 μl of the sample was removed, dried under nitrogen, and dissolved in 200 μl of water. For the amino acid derivatization, 50 μl of pretreated sample was mixed with 50 μl of protein precipitation reagent (containing L-norvaline as an internal calibration standard) and centrifuged at 13,200 rpm for 4 min. An aliquot of 10 μl of supernatant was removed and mixed with 50 μl of borate buffer (0.1 M, pH 8.8), after which the sample was mixed with 20 μl of derivatization solution (6-aminoquinolyl-*N*-hydroxysuccinimidyl carbamate) and incubated at 55°C for 15 min. After derivatization, the sample was cooled to 4°C and 50 μl samples were analyzed by LC-MS/MS.

### LC-MS/MS

LC-MS/MS was performed using a 3200 QTRAP^®^ LC-MS/MS System (Thermo Fisher Scientific, Waltham, MA, United States) using a previously published protocol (Joyce et al., 2016). LC was conducted with a 45 + AA-C18 column (150 × 4.6 mm, 5 μm) (MS Lab, Beijing, China) at 50°C with a flow rate of 1 ml/min and an injection volume of 5 μl. Mobile phase A consisted of 0.1% formic acid in water and mobile phase B was 1‰ formic acid in acetonitrile. The gradient consisted of 10% B for 0.1–1 min; 10% B to 70% B over 1–12 min; 70% B to 100% B over 12–12.1 min; 100% B from 12.1 to 15 min; 100% B to 10% B over 15–15.1 min; and 10% B from 15.1 to 20 min. For MS/MS, the parameters were: electrospray ionization source set in positive mode, spray voltage at +5500 V, GS1 (atomization gas) at 55 psi, GS2 (auxiliary gas) at 60 psi, scan mode in multiple reaction monitoring, collisionally activated dissociation at medium (collision gas), atomization temperature at 500°C, CUR (air curtain gas) at 20 psi, CXP (ejection voltage of collision chamber) at 2.0 V, and EP (injection voltage) at 10 V.

### Quantification of UAAs

Acquisition and processing of amino acid and metabolite data were performed with Analyst LC-MS/MS software version 1.5.1 (Thermo Fisher Scientific). An amino acid standard solution (MS Lab) was used to quantify the compounds, and the amino acid concentration were also calibrated using creatinine. The specific formula describes: calibrated UAA concentration (μmol/g creatinine) = UAA concentration (μmol/L)/creatinine concentration (mg/dL).

### Quality Control

For quality control of the LC-MS/MS method, we randomly selected two specimens and repeated the LC-MS/MS test once, and using the Pearson correlation analysis to determine the stability of the two tests (Supplementary Figure S1). To determine if there are Outliers, we first carried out the quality control of the initial data, then using principal component analysis to determine if there are Outliers. To determine if the sample is degraded, the sample with high Ammonia Level (NH4), or the ratio of glutamine/glutamic acid is too high, is considered to be sample degradation, the criterion was 3σ criterion, which is the mean + 3 standard deviation of all samples.

### Bioinformatics and Statistical Analyses

Group differences in clinical characteristics were evaluated by Student’s *t*-test (age) and Fisher’s exact test (sex) using the t.test and Fisher.test functions, respectively, in R package. The effects of clinical phenotypes on urine metabolomics was assessed by permutational multivariate analysis of variance using distance matrices (PERMANOVA) using the adonis function in the vegan R package. To assess the ability of the urine metabolomics data to distinguish between ASD and TD children, we performed sparse partial least squares discriminant analysis (PLS-DA) performed with the plsda function of the mixOmics package. The ability of UAA indicators to distinguish between ASD and TD children was evaluated using the Wilcoxon rank-sum test with the wilcox.test function in R package. Deseq2 in R package was used to calculate the fold differences between groups. Significantly different UAAs were selected according to a false discovery rate-corrected *p*-value of < 0.05 and a log2 fold change of >0.26. Finally, to identify UAA indicators with the potential for use as disease biomarkers, the 21 compounds identified from the discovery stage were used to train a support vector machine model using the e1071 function in R package. The optimal training UAA markers were validated using the pROC function in R package, and the area under the curve was calculated.

## Results

**FIGURE 1:**
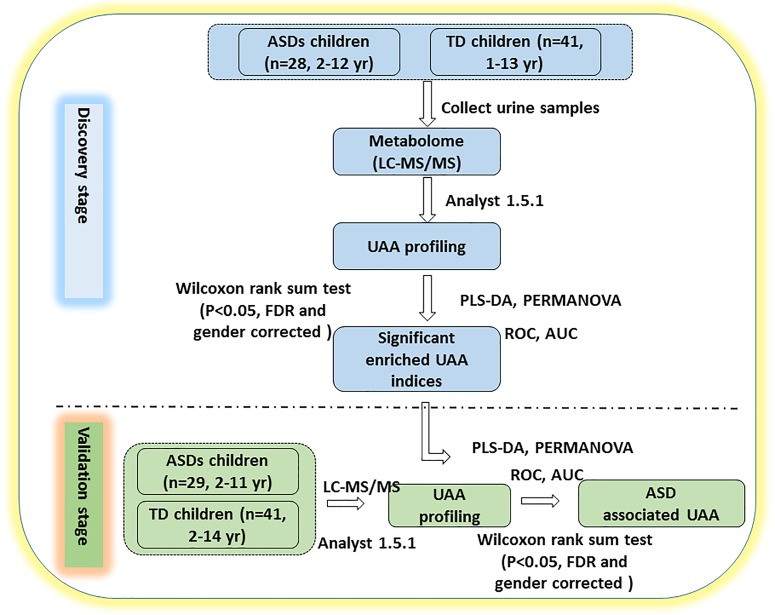
Study design. The study was performed in two stages. In the discovery stage, urine samples were collected and profiled for UAAs and related metabolites by LC-MS/MS. PLS-DA and PERMANOVA were applied to determine which of the indicators could distinguish between ASD and TD children. In the validation stage, additional urine samples were collected to verify whether the identified UAAs and metabolites could be potential biomarkers for the diagnosis of ASD. AUC, area under the receiver operating characteristic curve; FDR, false discovery rate; ROC, receiver operating characteristic. See text for all other abbreviations.

This study was carried out in two stages, as shown in Figure 1. Urine samples were collected from 28 ASD and 41 TD children for the discovery stage and from an additional cohort of 29 ASD and 41 TD children for the validation stage. The ASD and TD children were age- and sex-matched in the discovery cohort, but the validation cohort included significantly more males in the ASD group (27/29, 93.1%) than the TD group (7/41 [29.2%], *p* = 0.00212; Table 1 and Supplementary Table S1).

**Table 1:** Characteristics of study participants.

	Discovery cohorts (*n* = 69)			Validation cohorts (*n* = 70)		
Cohorts	ASD	TD	*p*-value	ASD	TD	*p*-value
Number	28	41	NA	29	41	NA
Male (n, %)	26, 92.8%	30, 73.2%	0.05928	27, 93.1%	7, 29.2%	0.00212
Age (mean ± SD, range) yr	5.4 ± 2.4, 2–12	5.1 ± 2.7, 1–13	0.73065	5.3 ± 2.2, 2–11	5.1 ± 2.5, 2–14	0.6358


*ASD, autism spectrum disorders; NA, not available; SD, standard deviation; TD, typical development; yr, years.*

**FIGURE 2:**
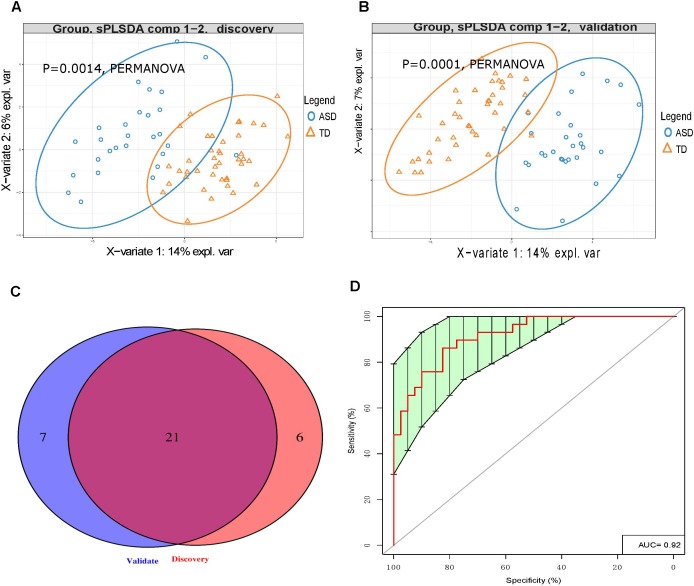
Urinary amino acid profiling distinguishes between ASD children and TD children. **(A,B)** PLS-DA analysis of discovery **(A)** and validation **(B)** samples showing that UAA indicators can clearly distinguish between ASD and TD children (*p* = 0.0014 and *p =* 0.0001, respectively, by PERMANOVA). **(C)** Venn diagrams showing the number of UAA indicators identified during the discovery and validation stages. **(D)** Receiver operating characteristic curve analysis of 7 UAA indicators identified by SVM model training as best able to distinguish between ASD and TD children.

LC-MS/MS analysis of the discovery cohort urine samples identified a total of 44 UAAs, 10 UAA indices, and 9 UAA-related metabolites (Supplementary Table S2). This dataset was analyzed by PLS-DA and we found that the ASD and TD children could be clearly distinguished (*p* < 0.0014; Figure 2A). The effect of gender and age on UAA were evaluated by PERMANOVA, the results showed that both the effect of gender and age on UAA were not significant (*p* = 0.3422 and *p* = 0.9267 for gender and age, respectively, Supplementary Table S3). Among the 63 UAA indicators identified, 27 were present at significantly different concentrations in the urine samples from ASD children compared with TD children; of these 27, 15 were decreased and 12 were increased in the ASD group compared with the TD group, it is worth noting that most (24 of 27) of the differences in UAA indicators were also significantly after adjusting for gender (Supplementary Table S4).

Similarly, LC-MS/MS analysis of the validation stage urine samples could readily discriminate between the 29 ASD children and 41 TD children (*p* < 0.0014, Figure 2B). Given that the gender ratio in the ASD and TD groups was not matched in the validation cohorts (*p* = 0.00212), and that the PERMANOVA results showed that gender had a significant impact on UAA (*p* = 0.0087, Supplementary Table S5), gender was considered as a covariate. In this analysis, a total of 28 UAA indicators were significantly different between the two sample groups, with 15 being present at lower levels and 13 at higher levels in the ASD group compared with the TD group, the same with the discovery stage, we found that most (23 of 28) of the differences in UAA indicators were also significantly after adjusting for gender (Supplementary Table S6).

Analysis of the discovery and validation cohort datasets identified 21 UAA indicators that were significantly different between the ASD and TD groups in both cohorts (Supplementary Table S7), and most (16 of 21) of the differences in UAA indicators were also significantly after adjusting for gender (Figure 2C and Table 2). Ten of these were significantly higher increased in the ASD compared with the TD group: methionine sulfoxide (MetS), homoarginine (Harg), 2-aminoisobutyric acid (2Aib), 3-methyl-histidine (3MHis), creatinine (Cr), arginine (Arg), arginine/ornithine (Arg/Orn) ratio, ornithine/citrulline (Orn/Cit) ratio, 5-hydroxytryptamine (5HT), and 4-hydroxyproline (Hyp), all increased UAA indicators, except for 2Aib, were significantly after adjusting for gender. An additional 11 UAA indicators were significantly decreased in the ASD group compared with the TD group: lysine (Lys), threonine (Thr), carnosine (Car), proline (Pro), ethanolamine (EtN), homocysteine (Hcy), α-aminoadipic acid (Aad), citrulline (Cit), anserine (Ans), 5-aminovaleric acid (5Ava), and aspartic acid (Asp), all decreased UAA indicators, except for Ans, were significantly after adjusting for gender.

**Table 2:** Urinary amino acids and related metabolites significantly different in ASD compared with TD children.

Description	Unit	Related pathways	TD.Mean	TD.Sd	ASD.Mean	ASD.Sd	FDR-corrected *P*-value	log2(FoldChange)	logit.p(gender adjusted)
5Ava	μM/g Cr	Lysine metabolism	0.01244213	0.013673155	0.004313431	0.00452196	1.047E-05	–1.25797285	9.68167E-05
Aad	μM/g Cr	Lysine metabolism	0.29428445	0.16953557	0.133094086	0.095259216	1.16368E-09	–0.95909614	3.81377E-07
Lys^∗^	μM/g Cr	Lysine metabolism	1.01745904	0.527912403	0.63389226	0.644147463	7.81929E-09	–0.57787983	0.000535586
Hcy	μM/g Cr	Methionine Cycle	0.02338386	0.014238709	0.011605619	0.010140154	2.73313E-07	–0.78929239	4.12653E-05
MetS	μM/g Cr	Methionine Cycle	0.00900822	0.010166376	0.074191197	0.067838536	1.46438E-10	3.028166086	3.12581E-06
2Aib^∗^	μM/g Cr	Others	0.00209377	0.011906901	0.002455312	0.003127731	6.50089E-12	1.548449365	0.312290372
3MHis	μM/g Cr	Others	0.34408042	0.424732786	0.784816004	0.436303948	3.09458E-08	1.125522347	3.16588E-06
Cr	mg/dL	Others	0.0952323	0.102476826	0.198102002	0.153653738	1.52052E-05	1.182416192	5.79771E-05
EtN	μM/g Cr	Others	3.90618985	1.595605519	2.178631635	1.053959589	1.62764E-10	–0.68737061	4.57777E-07
Thr	μM/g Cr	Others	0.9671388	0.370888994	0.676926102	0.220474256	5.20506E-09	–0.37250913	5.06616E-07
Ans	μM/g Cr	Oxidative Stress	0.06597364	0.141381394	0.033919983	0.178676624	1.75282E-05	–1.48244293	0.191276713
Car	μM/g Cr	Oxidative Stress	0.35545303	0.377437102	0.190996406	0.223686245	0.000220759	–0.66109471	0.001873569
5HT^∗^	μM/g Cr	Tryptophan metablism	0.0062606	0.003532686	0.012646994	0.01096659	3.61586E-08	1.121176777	1.83097E-06
Arg	μM/g Cr	Urea cycle	1.38267127	1.010837171	2.657701538	1.617449246	4.58364E-07	1.060903344	1.27669E-05
Arg/Orn^∗^	Ratio	Urea cycle	0.0562254	0.062053047	0.160024049	0.145571992	5.20506E-09	1.641541639	4.78869E-06
Asp^∗^	μM/g Cr	Urea cycle	0.11769798	0.43351078	0.020582196	0.029914952	2.14813E-10	–1.54575677	0.000126173
Cit	μM/g Cr	Urea cycle	0.08962332	0.156508192	0.034044216	0.029187287	3.09458E-08	–0.94952965	1.90847E-06
Harg	μM/g Cr	Urea cycle	0.03907258	0.030942833	0.095102036	0.070461315	4.97527E-05	1.384907093	6.01464E-06
Hyp^∗^	μM/g Cr	Urea cycle	0.11668983	0.039542028	0.149919338	0.051821658	0.000263407	0.501767958	0.000578734
Orn/Cit	Ratio	Urea cycle	0.00340283	0.00329391	0.020124928	0.070806444	4.58364E-07	1.741197074	0.000115062
Pro^∗^	μM/g Cr	Urea cycle	0.08375663	0.059400034	0.049528132	0.048022121	7.21531E-07	–0.57096039	0.001612036


*^∗^UAA indicators showing the best performance in the SVM model to distinguish between ASD and TD children; sd, Standard Deviation; log2(FoldChange) was determined using Deseq2; FDR, false discovery rate.*

Eight of the 21 significantly different UAA indicators were associated with the urea cycle, of which 5 (Orn/Cit, Arg/Orn, Harg, Arg, and Hyp) were present at higher levels and 3 (Pro, Cit, and Asp) were present at lower levels in the ASD group than the TD group. Hcy and MetS, both of which are methionine-associated metabolites, were present at lower and higher concentrations, respectively, in urine samples from the ASD children compared with TD children. Five additional UAA indicators that were lower in the ASD group than the TD group were two oxidative stress-related metabolites (Car and Ans) and three related to lysine metabolism (Lys, Aad, and 5Ava). Finally, 2Aib, Cr, 3MHis, and the tryptophan derivative 5HT were all increased and Thr and EtN were decreased in urine samples from ASD children compared with TD children (Table 2).

To identify the UAA indicators that most effectively distinguished between ASD and TD children, we performed support vector machine (SVM) training and constructed receiver operating characteristic curves for the 21 UAA indicator dataset. This analysis identified Lys, Pro, 5HT, Hyp, Arg/Orn, Asp, and 2Aib as the indicators best able to discriminate between ASD and TD children in the SVM model (area under the curve 0.92; Figure 2D). These metabolites might therefore be useful as biomarkers for the diagnosis of ASD.

## Discussion

### Abnormalities in the Ornithine Cycle in Children With ASD

The ornithine (or urea) cycle promotes the conversion of toxic ammonia molecules to non-toxic urea for subsequent excretion in the urine (Morris, 2002). Arg, Orn, and Cit are the key amino acids in this cycle. Consistent with the results of Anwar et al. (2018), we found that Arg concentrations were significantly higher in urine samples from ASD children than TD children. However, Arg, Orn, and Cit levels were present in decreasing concentrations in the urine of ASD children, suggesting the possibility that dysfunction of both arginase (Arg → Orn) and ornithine transcarbamylase (Orn → Cit) may lead to the observed abnormalities in the Orn/urea cycle in these children.

Perturbation of Orn/urea cycle function may result in the accumulation of ammonia in the blood of children with ASD. This possibility is supported by our finding that urinary Pro levels were significantly lower and Hyp levels were significantly higher in ASD compared with TD children. Pro conversion to the Orn/urea cycle intermediate Δ1-pyrroline-5-carboxylate by pyrroline-5-carboxylate reductase has previously been suggested to play a role in the clearance of blood ammonia levels (Wu, 1997; Baumgartner et al., 2000). Therefore, the combination of reduced Pro levels and increased conversion of Pro to Hyp may exacerbate the accumulation of ammonia. Although there have been relatively few recent studies of blood ammonia levels in children with ASD, some earlier studies provide clues of abnormalities (Cohen, 2002; Wang et al., 2012). Cohen et al. found that elevated blood ammonia concentrations may decreased γ-aminobutyric acid (GABA) transaminase activity and thus lead to an accumulation of GABA (Cohen, 2002).

**FIGURE 3:**
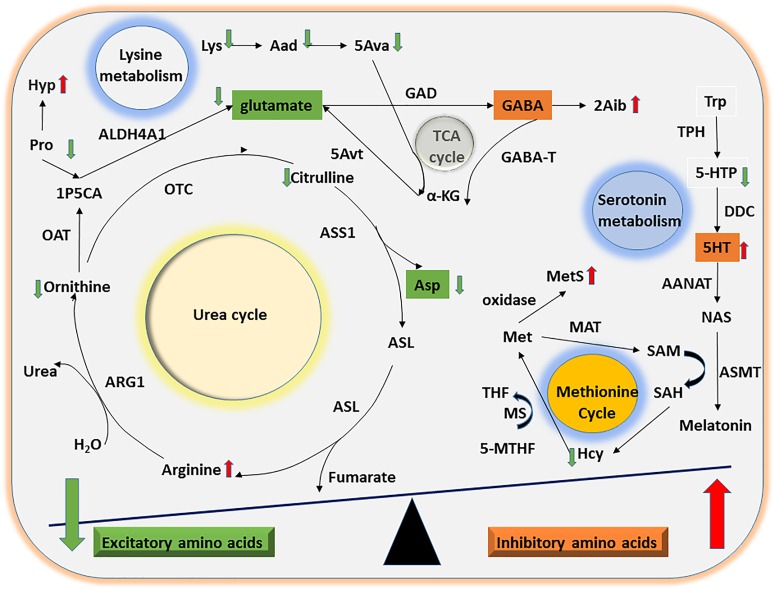
Mechanisms of UAA involvement in excitatory and inhibitory amino acid metabolism. The diagram shows the potential mechanisms by which the significant differences in UAA indicators identified between ASD and TD children might contribute to alterations in excitatory and inhibitory amino acid metabolism. Abnormalities in the Orn/urea and Met cycles and in Trp and Lys metabolism could lead to a decline in excitatory amino acids (e.g., glutamate and Asp) as well as a concomitant increase in inhibitory amino acids (e.g., 5HT).

Multiple lines of evidence indicate that imbalances in inhibitory and excitatory neurotransmitter levels are associated with the onset of ASD (Neale et al., 2012; O’Roak et al., 2012; Sanders et al., 2012). We found that urinary levels of the excitatory amino acid Asp were significantly lower in the ASD group than the TD group. Furthermore, Orn can be converted to the excitatory amino acid glutamate by ornithine aminotransferase and aldehyde dehydrogenase 4-A1, suggesting that a reduction in Orn concentrations may indirectly decrease glutamate levels. Taken together, our results suggest that abnormalities in the Orn/urea cycle may indirectly lead to an imbalance in the concentration of inhibitory and excitatory neurotransmitter amino acids in ASD children (Figure 3).

### Abnormalities in the Methionine Cycle in Children With ASD

Considerable evidence suggests that abnormal methylation is an important risk factor for ASD (Ladd-Acosta et al., 2014; Behnia et al., 2015; Hannon et al., 2018). The methionine cycle plays a significant role in generating intermediates that act as methyl group donors *in vivo* (Finkelstein, 1990). In the present study, we found that ASD children had abnormal urinary concentrations of two methionine cycle intermediates, Hcy and MetS, which were lower and higher, respectively, compared with the samples from TD children. Free methionine is converted to active methyl *S*-adenosylmethionine by methionine adenosyltransferase. Since Hcy, a sulfur-containing amino acid, can only be generated by conversion from methionine via *S*-adenosylmethionine and *S*-adenosylhomocysteine, the abnormally high levels of MetS and low levels of Hcy detected here strongly suggest that ASD children may have an abnormality in the methionine cycle (Figure 3).

### Evidence of High Oxidative Stress Levels in Children With ASD

Oxidative stress caused by increased free radical production and impaired antioxidant function is an important risk factor for autism (Damodaran and Arumugam, 2011). In the present study, we found that urinary Car and Ans levels were significantly lower in ASD children compared with TD children. Car, a dipeptide consisting of β-alanine and L-histidine, and Ans (β-alanyl-1-methyl-L-histidine) are functionally similar and act as antioxidants by capturing oxygen free radicals. Our results are consistent with those of Damodaran and Arumugam (2011), who found that oxidative stress markers were significantly higher in urine samples from a group of 45 ASD children compared with a matched group of 50 TD children. Similarly, Yui et al. (2017) found that ASD children had reduced levels of total urinary antioxidant levels that exceeded the oxidant levels.

### Abnormalities in 5HT Metabolism in Children With ASD

5-HT, also known as serotonin, is an inhibitory monoamine neurotransmitter derived from tryptophan (Knott and Curzon, 1972). Interestingly, 5HT was one of the earliest reported biomarkers of ASD, and several studies have shown that ∼25% of children with ASD have significantly elevated blood levels of 5HT (Muller et al., 2016). However, whether urinary 5HT levels could be used as an ASD biomarker is a controversial topic. Hérault et al. (1993) examined monoamine concentrations in urine samples from 23 children with ASD and 59 matched TD children, and they detected higher 5HT levels in the ASD group. However, Gevi et al. (2016) found that 5HT concentrations were significantly lower in urine samples from 30 children with ASD compared with 30 matched controls. Thus, our finding that ASD children have elevated urinary 5HT levels is consistent with the results of Hérault et al. (1993).

### Abnormalities in Lysine Metabolism in Children With ASD

Lysine is an essential amino acid that is easily destroyed during processing. We found that concentrations of Lys and its downstream metabolites Aad and 5Ava were significantly lower in urine samples from ASD children than TD children. Upon formation from Lys and Aad, 5Ava enters the tricarboxylic acid cycle to generate α-ketoglutaric acid and then glutamate via 5-aminovalerate transaminase (Figure 3). Thus, the abnormal Lys metabolism in children with ASD could indirectly lead to an imbalance in concentrations of the excitatory amino acid glutamate.

### Abnormal Metabolism of Other Amino Acids in Children With ASD

We found that the group of ASD children had significantly lower levels of EtN in their urine compared with the TD group. EtN is a precursor for the synthesis of the excitatory neurotransmitter acetylcholine, which also requires Met for *de novo* synthesis. The combination of decreased EtN concentration and abnormal Met metabolism may thus indirectly lead to a reduction in acetylcholine levels. We also detected that the ASD children had significantly higher levels of urinary Cr compared with TD children, which is consistent with the findings of Lussu et al. (2017). 2Aib, another UAA indicator found to be significantly elevated in the urine of children with ASD, is structurally similar to GABA and can also cross the blood-brain barrier (Ennis et al., 1994). Although the function of 2Aib is currently unknown, it has been speculated to play a role similar to the inhibitory neurotransmitter GABA.

In the present study, we found that ASD children had significantly higher urinary levels of 3MHis compared with TD children. This contrasts with the findings of Mavel et al. (2013), who used two-dimensional-nuclear magnetic resonance imaging to investigate the metabolome and found significantly lower 3MHis levels in ASD children (Mavel et al., 2013). Our study also revealed that urinary Thr levels were significantly lower in the urine of children with ASD compared with TD children, which is consistent with the study by Ming et al. (2012). Since Thr can be generated by transformation of Asp, our finding that urinary Asp levels were lower in the ASD cohort provides a potential explanation for the decrease in Thr.

Considering that the number of male ASD children is significantly higher than females, this study reached 10:1 of male: female ratio, so it was difficult in accurate gender matching; we found that although gender had a significant impact on UAA, the vast majority (19/21) of the differences in UAA were significant after adjusting for gender, these results may imply that gender is not necessarily the main cause of UAA changes. Regarding age, our age range is 2–15 years old. Although the range is large, considering the age of ASD and TD is matched and the impact of age on UAA is limited, we did not consider it as a covariate.

## Conclusion

This study used a two-stage discovery–validation approach to identify urinary markers potentially associated with ASD. The observed differences in UAA indicators suggest that ASD children may have disorders of amino acid metabolism that could directly or indirectly induce an imbalance in excitatory and inhibitory amino acids, including aspartate, glutamate, 5HT, and GABA. We also identified a panel of 7 UAA indicators that are potential biomarkers for ASD.

A limitation of this study is that we focused only on UAAs and related metabolites. Large-scale validation studies will be necessary to determine whether the 7 identified UAA indicators could indeed be used as biomarkers for ASD. The results are expected to lay the foundation for the development of a novel diagnostic method to facilitate the early diagnosis of ASD and prompt initiation of treatment.

## Author Contributions

All authors designed and executed the study and wrote the manuscript. MW and WnZ designed the project and drafted the manuscript. WiZ and AL executed the experiments. FH performed bioinformatics analysis. LQ, HW, YW, XL, and CC performed samples collection and clinical evaluation.

## Conflict of Interest Statement

The authors declare that the research was conducted in the absence of any commercial or financial relationships that could be construed as a potential conflict of interest.

## Supplementary Material

Figure S1 Technical repeat results for two samples. **(a)** sample 609, pearson cor = 0.994; **(b)** sample 642, pearson cor = 0.999.

Table S1 Clinical information and LC/MS MS result of study participants.

Table S2 Sixty-three UAA indicators.

Table S3 PERMANOVA results of discovery stage samples.

Table S4 Twenty-seven differences UAA indicators identified in discovery stage.

Table S5 PERMANOVA results of validate stage samples.

Table S6 Twenty-eight differences UAA indicators identified in discovery stage.

Table S7 Twenty-one differences UAA indicators identified in two stage.

## Abbreviations

ASD: autism spectrum disorder
LC-MS/MS: liquid chromatography-tandem mass spectrometry
TD: typical development
UAA: urinary amino acid
