# Supplementary figures and images for: Altered Urinary Amino Acids in Children With Autism Spectrum Disorders
Source: Front Cell Neurosci. 2019 Jan 25;13:7. doi: 10.3389/fncel.2019.00007 (PMC6354128; doi:10.3389/fncel.2019.00007)

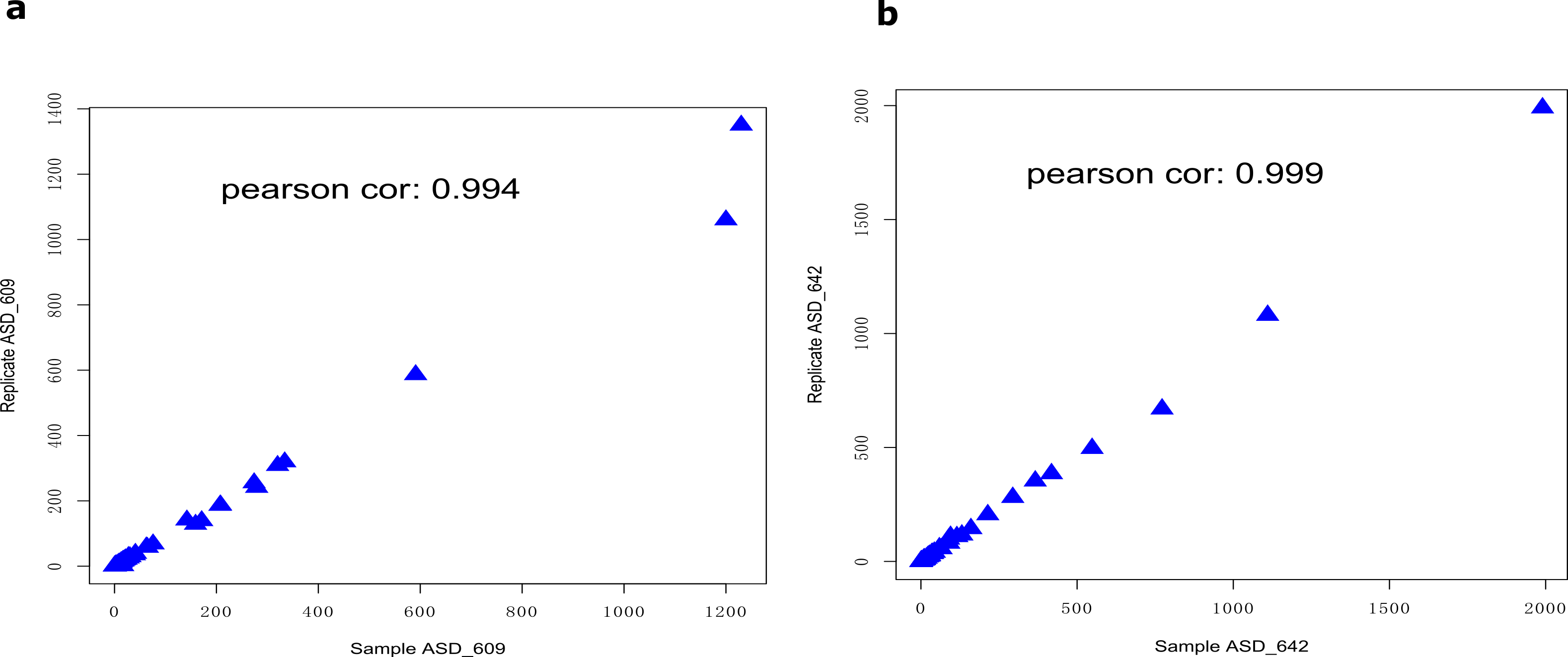

Supplement: Figure S1 — Technical repeat results for two samples. (a) sample 609, pearson cor = 0.994; (b) sample 642, pearson cor = 0.999. [file Image_1.PNG]
